# Hypericin-Mediated Photodynamic Therapy for Head and Neck Cancers: A Systematic Review

**DOI:** 10.3390/biomedicines13010181

**Published:** 2025-01-13

**Authors:** Jakub Fiegler-Rudol, Natalia Zięba, Radosław Turski, Maciej Misiołek, Rafał Wiench

**Affiliations:** 1 Department of Periodontal Diseases and Oral Mucosa Diseases, Faculty of Medical Sciences in Zabrze, Medical University of Silesia, 40-055 Katowice, Poland; rwiench@sum.edu.pl; 2 Department of Otorhinolaryngology and Laryngological Oncology in Zabrze, Medical University of Silesia, 41-800 Zabrze, Polandmaciej.misiolek@sum.edu.pl (M.M.); 3 Individual Dental Practice, Ul. Żarecka 128, 42-208 Częstochowa, Poland; turski.stomatologia@interia.pl

**Keywords:** hypericin, photodynamic therapy, head and neck cancer, apoptosis, immunomodulation, cytokines

## Abstract

**Background**: Conventional treatments for cancers of the head and neck region are often associated with high recurrence rates and impaired quality of life. Photodynamic therapy (PDT) has emerged as a promising alternative, leveraging photosensitizers such as hypericin to selectively target tumour cells with minimal damage to surrounding healthy tissues. **Objectives**: We aimed to evaluate the efficacy and underlying mechanisms of hypericin-mediated PDT (HY-PDT) in treating head and neck cancers. **Methods**: Adhering to PRISMA 2020 guidelines, a systematic search was conducted across PubMed/Medline, Embase, Scopus, and the Cochrane Library for studies published between January 2000 and December 2024. Inclusion criteria encompassed preclinical in vitro and in vivo studies and clinical trials focusing on HY-PDT for head and neck malignancies and its subtypes. **Results**: A total of 13 studies met the inclusion criteria, comprising both in vitro and in vivo investigations. HY-PDT consistently demonstrated significant cytotoxicity against squamous cell carcinoma cells through apoptotic and necrotic pathways, primarily mediated by ROS generation. Hypericin exhibited selective uptake in cancer cells over normal keratinocytes. Additionally, HY-PDT modulated the tumour microenvironment by altering cytokine profiles, such as by increasing IL-20 and sIL-6R levels, which may enhance antitumor immunity and reduce metastasis. **Conclusions**: HY-PDT emerges as a highly promising and minimally toxic treatment modality for head and neck cancers, demonstrating efficacy in inducing selective tumour cell death and modulating the immune microenvironment. Despite the encouraging preclinical evidence, significant methodological variability and limited clinical data necessitate further large-scale, standardized and randomized controlled trials.

## 1. Introduction

### 1.1. Rationale

Head and neck cancers represent a heterogeneous group of malignancies in anatomically complex and functionally critical regions, such as the oral cavity, pharynx, and larynx [1]. These cancers pose significant clinical and therapeutic challenges, often leading to impaired speech, swallowing difficulties, and disfigurement, substantially affecting patients’ quality of life [2]. Conventional treatment options, such as surgery, radiation therapy, and chemotherapy, although often effective at controlling or reducing the burden associated with tumours, are frequently associated with long-term complications, high recurrence rates, and considerable morbidity [3]. Consequently, there is an ongoing need to explore more selective, and less invasive, treatments that can improve clinical outcomes and maintain or restore function for individuals suffering from these diseases [4]. Photodynamic therapy (PDT) has emerged as a promising treatment for various cancers, including those of the head and neck region [5]. PDT involves the administration of a photosensitizing agent that preferentially accumulates in tumour cells, followed by irradiation with a specific wavelength of light [6]. This interaction generates reactive oxygen species (ROS) that cause oxidative damage to cellular components, ultimately leading to tumour cell death [6]. Moreover, beyond the direct cytotoxic damage resulting from ROS generation, PDT also exerts its therapeutic effects by acting on the tumour vasculature that causes vascular shutdown, reduced blood flow, and consequent ischemic cell death and by triggering a range of immunomodulatory mechanisms. These include the release of tumour-associated antigens, recruitment of immune effector cells, and alteration of cytokine profiles, all of which can collectively enhance systemic anti-tumour immunity [7].

Unlike traditional cancer treatments, PDT offers the advantage of spatial selectivity—light can be directed precisely to the tumour site—thereby minimizing harm to surrounding healthy tissues [4,5,6]. Additionally, PDT has been reported to provoke immunomodulatory effects, potentially enhancing anti-tumour immunity and reducing the likelihood of metastasis and recurrence [7]. Within the spectrum of available photosensitizers, hypericin has gathered significant attention for its unique chemical and photophysical properties [8,9]. Brancaleon and Moseley have demonstrated that endoscopy, combined with light irradiation, can effectively excite photosensitizers [10]. Lasers are widely used for superficial and interstitial PDT due to their monochromatic nature, coherent light, high optical power, and ability to tune wavelengths to match specific photosensitizers [11]. Their narrow, collimated beams are often coupled with optical fibres for endoscopic or interstitial applications [11].

Hypericin is a naturally occurring compound primarily isolated from Hypericum perforatum (St. John’s Wort), a plant with a long history of therapeutic applications [8]. Its ability to accumulate within cellular compartments, such as mitochondria and the endoplasmic reticulum, makes it highly effective in disrupting critical cellular functions [9]. Preclinical studies have shown that hypericin-based PDT can induce apoptosis, necrosis, and other forms of cell death in malignant cells [12]. Additionally, hypericin-mediated PDT (HY-PDT) has demonstrated potential advantages over certain conventional treatments, including reduced systemic toxicity and a more favourable safety profile [13]. As research into HY-PDT has grown, investigations have extended into its immunomodulatory effects. Beyond direct cytotoxicity, hypericin’s photoactivated state may influence cytokine production, affect tumour-associated immune cells, and modulate inflammatory responses [14]. These immunological alterations could enhance tumour control, reduce metastatic potential, and improve long-term clinical outcomes [14,15,16,17,18].

### 1.2. Objectives

The primary aim of this systematic review is to provide a synthesis of the existing literature on HY-PDT for head and neck cancers. We seek to determine the efficacy of HY-PDT in reducing tumour burden as well as improving survival. It is important to also explore the underlying mechanisms of action, including the generation of ROS and subsequent immunological responses that may contribute to enhanced therapeutic outcomes as well as to compare HY-PDT to alternative or established therapies, evaluating its potential clinical applications. In doing so, this review will identify knowledge gaps, highlight areas for future research, and provide evidence-based guidance for clinicians and researchers aiming to advance the treatment of head and neck cancers through HY-PDT.

## 2. Materials and Methods

### 2.1. Focused Question and Null Hypothesis

A systematic review was conducted using the PICO framework to evaluate the efficacy of hypericin-mediated photodynamic therapy for head and neck cancers [19]. The research focused on patients diagnosed with cancer of the head and neck region (Population). The review examined whether treatment with HY-PDT (Intervention), in comparison with other approaches such as light irradiation alone, use of hypericin without light activation, or alternative cancer therapies (Comparison), results in improved tumour reduction, immunomodulatory effects, or cell viability reduction (Outcome). The null hypothesis is that there is no significant difference in the effectiveness of HY-PDT compared with alternative treatments for reducing tumour size, modulating the immune response, or inducing cellular apoptosis.

### 2.2. Search Strategy

This systematic review, registered in the PROSPERO database under ID CRD42024627236, was conducted in adherence to the PRISMA 2020 guidelines [20]. A comprehensive electronic search was carried out across PubMed/Medline, Embase, Scopus, and the Cochrane Library (details of search terms provided in Table 1). Three authors (J.F.-R., N.Z. and R.T.) independently searched four databases using predefined standardized terms. The results were filtered to include studies published in English between 1 January 2000 and 3 December 2024. The authors chose to include older studies, as their conclusions were deemed relevant to this day and no similar research had been carried out since that time. Initial screening was performed by reviewing titles and abstracts in order to identify studies meeting the inclusion criteria (outlined in Table 2). The inclusion and exclusion criteria for study selection were rigorously applied to ensure the relevance and quality of the reviewed literature. Subsequently, two authors (J.F.-R. and N.Z.) conducted an in-depth review of the full texts to extract relevant data. A snowballing technique was also applied, examining reference lists of eligible articles to identify additional relevant studies, but no additional articles were found.

### 2.3. Selection of Studies

In the study selection phase of this systematic review, three reviewers (J.F.-R., N.Z. and R.W.) conducted independent assessments of the titles and abstracts from the identified articles to reduce potential bias. Discrepancies in study eligibility were addressed through thorough deliberation among the reviewers until consensus was reached. By following a process aligned with the PRISMA guidelines, the review prioritized the inclusion of highly relevant and methodologically robust studies, enhancing the overall reliability and replicability of the findings [20].

### 2.4. Risk of Bias in Individual Studies

In the initial phase of study selection, three reviewers (J.F.-R., N.Z. and M.M.) independently assessed the titles and abstracts of the identified articles to mitigate potential bias. To ensure consistency in evaluations, inter-reviewer agreement was quantified using Cohen’s kappa statistic [21]. Any conflicts regarding the inclusion or exclusion of studies were resolved through comprehensive discussions among the authors, ultimately reaching a unanimous consensus.

### 2.5. Quality Assessment

The quality of the included studies on HY-PDT for head and neck carcinomas was independently assessed by three reviewers (J.F.-R., N.Z. and M.M.). The evaluation focused on critical aspects of PDT design, execution, and reporting, specifically regarding the use of hypericin. The objectivity and reproducibility of the results were prioritized. The risk of bias was assessed using the following criteria, with a score of 1 for “yes” and 0 for “no”, as follows:

1. Was the specific concentration of hypericin as the photosensitizer clearly indicated?
2. Was the origin or source of the hypericin provided?
3. Was the incubation time for the hypericin clearly stated?
4. Were detailed light source parameters (type, wavelength, energy density, fluence, and power density) reported?
5. Was a power meter used to verify the light parameters?
6. Was a negative control group included in the experimental design?
7. Were numerical results reported with relevant statistical analyses?
8. Was there a clear method for addressing missing outcome data?
9. Was the study free from potential conflicts of interest related to its source of funding?

Studies were categorized based on the total number of affirmative (“yes”) responses to the following criteria: high risk of bias: 0–3; moderate risk of Bias: 4–6; low risk of bias: 7–9. Each study’s results were analysed to assign a corresponding bias classification—low, moderate, or high. The methodology for assessing the risk of bias adhered to the recommendations outlined in the *Cochrane Handbook for Systematic Reviews of Interventions* [22]. This framework ensures a comprehensive and systematic evaluation of the included studies.

### 2.6. Risk of Bias Across Studies and Quality Assessment Presentation

Table 3 presents the results of the risk of bias assessment for each of the 13 studies included after full-text review. To qualify for inclusion, studies needed to have achieved a minimum score of 6. All selected studies were determined to have a low or moderate risk of bias, with three achieving the highest possible score of 9 [23,24,25].

### 2.7. Data Extraction

Once the selection of articles was finalized through consensus, three reviewers (J.F.-R., M.M. and R.W.) systematically extracted data on multiple parameters. These included citation details (author names and year of publication), study design, type of cancer examined, characteristics of the experimental and control groups, follow-up durations, reported outcomes, specifications of the light source, concentrations of hypericin, laser parameters, as well as details on incubation and irradiation durations.

## 3. Results

### 3.1. Study Selection

**Figure 1:**
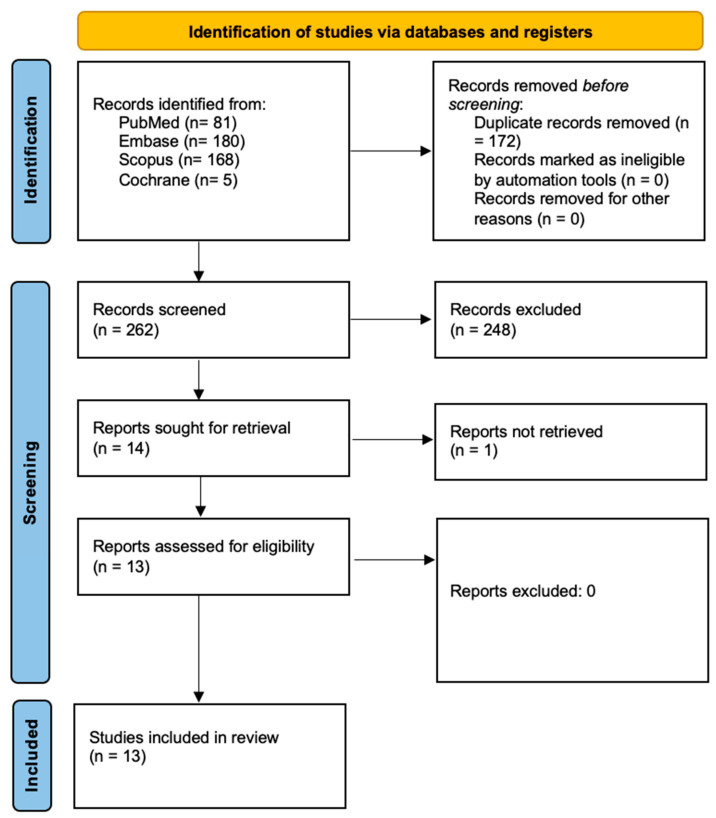
Prisma 2020 flow diagram [20].

Figure 1 outlines the research process conducted in accordance with the PRISMA guidelines [20]. The initial database search identified 434 articles, which were reduced to 262 after removing duplicates. Screening the titles and abstracts resulted in 14 studies being deemed eligible for full-text assessment. Of these, one study was excluded, as the full text had been removed by the authors. Ultimately, 13 studies, all published within the past 25 years, were included in the final analysis. A detailed summary of these studies is provided in Table 4.

### 3.2. General Characteristics of the Included Studies

Table 5, Table 6 and Table 7 provide a detailed summary of the data extracted from the studies that fulfilled the inclusion criteria and were incorporated into the review. The summaries include key information on the overall characteristics of the studies, technical details of the light sources utilized, and the properties of hypericin as photosensitizer in photodynamic therapy protocols.

### 3.3. Main Study Outcomes

HY-PDT has shown potential in cancer treatment due to its ability to generate ROS upon activation by specific wavelengths of light, leading to tumour cell death through apoptosis, necrosis, or both. Studies by Bhuvaneswari et al. (2007) explored hypericin’s efficacy in various cancer types, including nasopharyngeal carcinoma (NPC), head and neck squamous cell carcinoma, oral squamous cell carcinoma, melanoma, and other solid tumours [26]. With regard to NPC, Bhuvaneswari et al. have demonstrated that PDT triggers hypoxia within tumours, leading to vascular endothelial growth factor (VEGF) upregulation via the HIF-1α pathway. This effect can be mitigated with celebrex, a COX-2 inhibitor, which downregulates VEGF expression, potentially preventing tumour regrowth and enhancing PDT outcomes. Blank et al. (2001) highlighted HY-PDT’s effectiveness in inducing extensive tumour necrosis and inflammation in highly invasive solid tumours, although systemic immune antitumoral responses remain limited [27]. Research into the biodistribution and phototoxicity of hypericin, as investigated by Du et al. (2003), demonstrates its optimal activation at wavelengths around 593 nm, corresponding to its absorption peaks [29,30,31]. Hypericin accumulates predominantly in tumour cells, with localization in organelles such as the endoplasmic reticulum and Golgi apparatus [31]. Tumour regression is most effective when light irradiation is applied at peak tumour uptake times, such as six hours post-hypericin injection, as shown in Du et al. (2003) [29,30,31].

In vitro studies by Laffers et al. (2015) confirm hypericin’s ability to induce apoptosis at low concentrations and short light exposures, while in vivo applications show significant tumour regression in smaller tumours and partial responses in larger ones [34]. However, the therapy’s efficacy is challenged by limited light penetration, particularly in larger tumours, and potential off-target effects, as noted by Olek et al. (2023), who have reported cytotoxicity in healthy fibroblasts and keratinocytes [23]. PDT’s immunomodulatory effects have been highlighted by Olek et al. (2024), with reports of alterations in cytokine secretion [24]. For example, HY-PDT increases pro-inflammatory cytokines like IL-8 and IL-6 in cancer cells, while reducing immunosuppressive factors such as PTX3. Despite these effects, systemic immune responses remain underwhelming, with limited activation of antitumoral immunity, as suggested by Du et al. (2002) [29]. Furthermore, studies investigating hypericin-mediated PDT in cell lines, such as those by Xu et al. (2010), reveal varying responses depending on the tumour microenvironment, emphasizing the need for tailored therapeutic approaches [25]. Selectivity for cancer cells has been observed, particularly in melanoma and squamous cell carcinoma, where Wozniak et al. (2023) have demonstrated hypericin’s preferential accumulation in malignant cells [35]. However, hypericin’s poor solubility and sensitivity to environmental factors remain significant barriers to clinical application [35]. Advances in laser technology, combination therapies, and drug delivery systems, as noted by Sharma et al. (2012), are critical to addressing current limitations, such as suboptimal light penetration, off-target effects, and inadequate immune responses [33]. Future research, as recommended by Head et al. (2006) and others, must focus on optimizing dosing regimens, light parameters, and drug formulations to enhance therapeutic efficacy and minimize adverse effects, paving the way for hypericin-based PDT to become a more reliable and widely adopted cancer treatment modality [32]. Table 5 summarises the outcomes of each study.

Hypericin preferentially accumulates in tumours due to increased vascular permeability and active cellular uptake of hydrophobic molecules [30,31,35]. Studies show peak tumour uptake occurs hours post-injection, guiding irradiation timing to maximize tumour-to-healthy tissue concentration ratios [30,31,35]. Immune cells, especially neutrophils, may internalize hypericin in the tumour microenvironment, potentially enhancing antitumor activity or sensitizing them to oxidative stress [27]. Hypericin localizes to organelles like the ER, Golgi, and mitochondria, triggering apoptosis or necrosis [22,31,34]. Hypericin’s advantages over other photosensitizers include strong absorption peaks (545–595 nm), high singlet oxygen quantum yield, low dark toxicity, and immunomodulatory effects like cytokine profile modulation (e.g., IL-8, IL-20, TNF-α receptors) [24,27,28,35]. These features make hypericin promising for photodynamic therapy (PDT), particularly in head and neck cancers [28,35]. Hypericin retains characteristic absorption (~470, 545, 595 nm) and fluorescence (~590, 640 nm) spectra in cells and tumours, enabling fluorescence imaging for localization and efficacy monitoring [28,30,32]. Effective PDT is achieved with micromolar hypericin concentrations (0.5–5 µM) and moderate light fluences (3–20 J/cm^2^), favouring apoptosis over necrosis [25,34,35]. Excessive hypericin or light doses shift responses toward necrosis, highlighting the need for parameter optimization [34,35]. During hypericin-mediated PDT, reactive oxygen species, like singlet oxygen, superoxide, and hydroxyl radicals, induce oxidative damage in organelles, driving apoptosis or necrosis [28,31,35]. Hypericin-mediated PDT also disrupts tumour vasculature, creating transient hypoxia. Combining hypericin-mediated PDT with celecoxib blocks the COX-2/HIF-1α/VEGF pathway, mitigating hypoxia-driven VEGF rebound and improving tumour control [26].

### 3.4. Characteristics of Light Sources Used in PDT

There were significant differences in the protocols used by the studies included in this review. Table 6 compares the physical parameters of the light sources used in studies that satisfied the inclusion criteria. Table 7 compares the concentrations and incubation time of hypericin.

## 4. Discussion

### 4.1. Results in the Context of Other Evidence

This systematic review provides enough evidence to reject the null hypothesis. HY-PDT demonstrates significant preclinical promise as a selective and minimally toxic therapeutic option for head and neck cancers, showing efficacy in inducing tumour cell death (via apoptosis and necrosis), modulating the immune microenvironment, and offering potential advantages over conventional therapies. HY-PDT generates ROS that induces both apoptotic and necrotic cell death in head and neck cancer cells [25,26,34]. Cancer cells such as SCC-25 demonstrate enhanced sensitivity to hypericin compared with normal cells, emphasizing the selective cytotoxic potential of HY-PDT [31,33,35]. This therapy also influences the tumour microenvironment by altering cytokine production, including IL-8 and IL-20, which can modulate immune responses and impact tumour progression [23,24,29]. When combined with agents like celecoxib, HY-PDT can significantly downregulate pro-angiogenic factors such as VEGF, potentially preventing tumour regrowth [26,27]. Optimal therapeutic outcomes depend on the precise control of parameters, including wavelength, intensity, and duration of light exposure, while treatment efficacy decreases with larger tumour sizes due to limitations in light penetration [25,28,32,33]. Despite these challenges, HY-PDT maintains relatively localized cytotoxicity with minimal systemic toxicity, and its immunomodulatory effects—stemming from altered cytokine expression and influences on tumour-associated immune cells—further support its role as a promising treatment modality [23,24,27,30,34,35]. Mechanistically, hypericin localizes within cellular organelles such as mitochondria, causing substantial structural damage and promoting tumour cell death [28,34]. Although further clinical studies are necessary, the accumulating evidence suggests that HY-PDT holds considerable promise as a therapeutic approach for head and neck cancers [24,30,33]. Numerous studies have come to similar conclusions. Several studies have evaluated the application of PDT in other malignancies. Dong et al. have demonstrated that HY-PDT holds considerable potential as a precision cancer therapy by engaging molecular pathways to initiate apoptosis, necrosis, and autophagy. However, further inquiry is necessary in order to address obstacles, such as limited bioavailability, and to enhance its clinical implementation [36].

Kamuhabwa et al. have provided evidence that HY-PDT can trigger either apoptosis or necrosis in AY-27 urinary bladder carcinoma cells, depending on the concentration used. These findings suggest that hypericin-based photodynamic therapy may play a crucial role in treating superficial bladder carcinoma [37]. Zupko et al. have demonstrated that, in AY-27 tumours, the primary mechanism of HYP-PDT involves altering the tumour vasculature rather than causing direct cellular damage [38]. Huygens et al. have demonstrated that the combination of hypericin and hyperoxygenation can nearly eradicate RT-112 bladder cancer cells through apoptotic mechanisms [39]. In another study, Blank et al. investigated the impact of varying wavelengths in HYP-PDT for C26 colon carcinoma cells and found that in vitro irradiation of hypericin-sensitized cells reduced cell viability in a dose-dependent manner [40]. Ferenc et al. have reported that employing HYP-PDT together with genistein impaired the proliferation of MCF-7 and MDA-MB-231 cells and promoted their apoptosis [41]. Furthermore, Delaey et al. were the first to establish that hypericin-induced photocytotoxicity in HeLa cells is influenced by cell density, as sparse cultures displayed greater sensitivity to PDT compared with confluent cell layers [42]. Photodynamic therapy offers a means of controlling the proliferation of glioma cells, a notably invasive category of cancers. In a groundbreaking study, Miccoli et al. found that photoactivation of hypericin disrupted the energy metabolism of SNB-19 glioma cells by preventing hexokinase from anchoring to the mitochondria [43]. These findings indicate that hypericin may serve as a potent phototoxic agent against glioma tumours [43,44]. Lavie et al. have demonstrated that the photoactivation of HYP and dimethyl tetrahydroxyhelianthrone (DTHe) induces both apoptotic and necrotic cell death in HL-60 and K-562 cells, accompanied by nucleolar chromatin condensation [45]. Liu’s work has revealed that HYP-PDT markedly inhibits the proliferation of MiaPaCa-2 and PANC-1 pancreatic cancer cells both in vivo and in vitro, suggesting that hypericin-based photodynamic therapy could be an effective treatment strategy for this malignancy [46]. In related research, Chen et al. have shown that HYP-PDT can induce vascular damage and apoptosis within a radiation-induced fibrosarcoma-1 mouse tumour model [47]. These findings highlight the broad therapeutic promise of hypericin-based photodynamic therapy as a versatile and effective treatment modality against a wide spectrum of malignancies.

Beyond direct cytotoxicity, HY-PDT influences antitumor immunity by modulating inflammatory pathways. Studies report changes in cytokine profiles, such as IL-8 and soluble TNF receptors, in tumour microenvironments, impacting local inflammation and systemic immune responses [23,24,27,29]. While HY-PDT triggers tumour necrosis and inflammation, systemic immune activation remains limited, reflecting the complexity of immune responses across tumour models. These findings highlight HY-PDT’s ability to reshape tumour-related immune pathways, enhancing its therapeutic potential. Picosecond pulsed laser irradiation enhances HY-PDT efficacy compared with nanosecond pulses by increasing ROS generation and improving the subcellular targeting of organelles like mitochondria and the ER [28,34,35]. Shorter pulse durations enable deeper tissue penetration, reduce thermal diffusion, and minimize damage to healthy tissues, improving selectivity and safety. Picosecond pulses also amplify vascular disruption and immunomodulatory effects, further boosting antitumor immune responses.

Optimizing pulse duration is an important parameter when maximizing therapeutic outcomes in HY-PDT. Hypericin presents several advantages over conventional photosensitizers, such as photofrin and aminolevulinic acid in PDT, which include stronger absorption peaks, higher singlet oxygen quantum yields, lower dark toxicity, and selective tumour accumulation, and which increase cytotoxicity to the cells while minimizing off-target effects [28,31,34,35]. Additionally, hypericin’s immunomodulatory properties, such as cytokine modulation, contribute to antitumor immunity, unlike many traditional photosensitizers [23,24,27]. While hypericin faces challenges, like poor solubility, chemical modifications and delivery systems address these limitations, solidifying its role as a promising alternative in PDT [23,24,25,28,29,30,31,32,33,34,35]. Chemical modifications to hypericin improve solubility, stability, and targeting capabilities. Strategies include conjugation with antibodies or peptides for selective uptake, encapsulation in nanoparticles for controlled release, and PEGylation to enhance bioavailability [28,35].

### 4.2. Limitations of the Evidence

While the review synthesizes compelling evidence for HY-PDT, several limitations in the available literature were identified. Most studies are preclinical, with limited in vivo and clinical data, restricting the ability to generalize findings to human populations. Furthermore, methodological heterogeneity—such as variations in hypericin concentration, light source parameters, and treatment protocols—complicates direct comparisons and meta-analytic assessments. The relatively small sample sizes and short follow-up periods in many studies further constrain the ability to evaluate long-term outcomes, including survival rates and recurrence. Studies lacked comprehensive data on the safety profile and adverse effects of HY-PDT.

### 4.3. Limitations of the Review Process

The significant variability among the included studies necessitated a narrative approach to synthesizing the findings. Differences in study designs, intervention strategies, and outcome measures may have introduced bias into the overall evaluation of HY-PDT. Additionally, the substantial heterogeneity in parameters precluded the use of the GRADE framework, making it challenging to formulate robust, evidence-based recommendations. Future investigations should prioritize well-structured randomized controlled trials featuring direct comparisons of key parameters and standardized treatment protocols. Excluding non-English publications and grey literature likely narrowed the scope of this review, potentially omitting valuable insights. These constraints highlight the pressing need for more standardized and harmonized research to enable systematic comparisons and comprehensive quantitative analyses.

### 4.4. Implications for Practice, Policy, and Future Research

Clinicians may consider integrating HY-PDT into treatment protocols, especially for patients who are poor candidates for surgery or systemic therapies. However, careful parameter optimization is critical to ensure effective tumour targeting while minimizing harm to healthy tissues. Policymakers and professional organizations could consider developing preliminary guidelines for HY-PDT use in head and neck cancers. Such guidelines would benefit from standardized reporting of treatment parameters and outcomes, ultimately shaping future clinical trials and informing evidence-based policies that encourage broader adoption once safety and efficacy are conclusively demonstrated. Regulatory agencies and research bodies should support efforts to standardize HY-PDT protocols, including photosensitizer concentrations, light delivery methods, and treatment schedules. Establishing uniform standards will facilitate meaningful comparisons across studies, accelerate the generation of high-quality evidence, and streamline the regulatory approval process for HY-PDT-based interventions. Further research should focus on large-scale, multicentre, and well-controlled randomized clinical trials that incorporate consistent treatment parameters and long-term follow-up. It is important to note that most of these studies are preclinical, and implementing laser technology in the nasopharynx presents significant logistical challenges. Studies exploring combination strategies with immunomodulatory agents, improved photosensitizer formulations for enhanced tissue penetration, and advanced imaging techniques for treatment guidance will be critical. Additionally, investigating biomarkers predictive of response could help tailor HY-PDT to individual patient profiles, ultimately improving treatment efficacy and patient outcomes. While the reviewed studies have generally reported minimal systemic toxicity, the observed cytotoxicity to healthy fibroblasts and the potential for inflammatory responses from necrotic cell death underscore the need for careful parameter optimization and detailed adverse event reporting in future studies.

## 5. Conclusions

This systematic review highlights the considerable potential of hypericin-mediated photodynamic therapy as a promising treatment modality for head and neck cancers. The preclinical and limited clinical data indicate that HY-PDT exerts potent cytotoxic effects on malignant cells, mediated through the generation of reactive oxygen species and leading to both apoptotic and necrotic cell death. Notably, hypericin displays preferential uptake and toxicity toward cancer cells compared with normal keratinocytes, suggesting a favourable therapeutic index. Beyond its direct cytotoxicity, HY-PDT can modulate the tumour microenvironment by influencing cytokine profiles and inflammatory mediators, thus potentially enhancing antitumor immune responses and reducing the likelihood of recurrence. Additionally, combination strategies—such as co-administration with COX-2 inhibitors—may improve treatment outcomes by downregulating pro-angiogenic factors like VEGF, potentially inhibiting tumour regrowth. Despite these encouraging findings, several challenges must be addressed before HY-PDT can be widely integrated into clinical practice. A key limitation is the anatomy of the pharynx and the associated difficult access.

## Figures and Tables

**Table 1:** Search syntax used in the study.

Source	Search Term	Number of Results
PubMed/MEDLINE	((“Hypericin”) AND (“Photodynamic Therapy” OR “Photochemotherapy”) AND (“Squamous Cell Carcinoma” OR “Oral Cancer” OR “Carcinoma”) AND (“Keratinocytes” OR “Fibroblasts” OR “Cells”))	81
Embase	(‘hypericin’/exp OR ‘hypericin’) AND (‘photodynamic therapy’/exp OR ‘photodynamic therapy’ OR ‘photochemotherapy’/exp OR ‘photochemotherapy’) AND (‘squamous cell carcinoma’/exp OR ‘squamous cell carcinoma’ OR ‘oral cancer’/exp OR ‘oral cancer’ OR ‘carcinoma’/exp OR ‘carcinoma’) AND (‘keratinocyte’/exp OR ‘keratinocyte’ OR ‘fibroblast’/exp OR ‘fibroblast’ OR ‘cell’/exp OR ‘cell’)	180
Scopus	(TITLE-ABS-KEY(hypericin) AND (TITLE-ABS-KEY(“photodynamic therapy”) OR TITLE-ABS-KEY(photochemotherapy)) AND (TITLE-ABS-KEY(“squamous cell carcinoma”) OR TITLE-ABS-KEY(“oral cancer”) OR TITLE-ABS-KEY(carcinoma)) AND (TITLE-ABS-KEY(keratinocyte) OR TITLE-ABS-KEY(fibroblast) OR TITLE-ABS-KEY(cell))	168
Cochrane	(MH “Hypericin” OR “Hypericin”) AND (“Photodynamic Therapy” OR “Photochemotherapy” OR “Light Therapy”) AND (“Carcinoma” OR “Squamous Cell Carcinoma” OR “Cancer” OR “Oral Cancer” OR “Neoplasms”)	5

**Table 2:** Selection criteria for papers included in the systematic review.

Inclusion Criteria	Exclusion Criteria
Preclinical studies (e.g., in vitro, in vivo models). Clinical studies (e.g., randomized controlled trials, cohort studies). Review articles and meta-analyses relevant to PDT with hypericin or riboflavin. Studies addressing squamous cell carcinoma, including subtypes (oral squamous cell carcinoma, head and neck squamous cell carcinoma). Use of hypericin or riboflavin in photodynamic therapy. Cellular and molecular effects (e.g., apoptosis, necrosis, oxidative stress). Tumor response (e.g., reduction in tumor size, viability). Immunomodulatory effects. Articles published in English or Polish. Studies published within the last 25 years.	Gray literature Studies unrelated to PDT or those using different photosensitizers than hypericin or riboflavin. Non-original research, such as editorials or opinions without data. Studies focused on other cancer types, without relevance to the head and neck region. Animal or cell-line studies that do not include squamous cell carcinoma models. Lack of specific results on PDT efficacy or mechanisms. Articles with incomplete data, inaccessible full texts, or unpublished studies. Articles published in languages other than English or Polish, unless a translation is available.

**Table 3:** The results of the quality assessment and risk of bias across the studies.

Study	Question										
	1	2	3	4	5	6	7	8	9	Total	Classification
Bhuvaneswari et al. (2007) [26]	1	1	1	1	0	1	1	0	0	6	Moderate
Blank et al. (2001) [27]	1	0	0	1	0	1	1	0	1	5	Moderate
Bublik et al. (2006) [28]	1	1	1	1	1	1	1	0	1	8	Low
Du et al. (2002) [29]	1	1	1	1	1	0	1	1	1	8	Low
Du et al. (2003) [30]	1	1	1	1	1	0	1	1	1	8	Low
Du et al. (2004) [31]	1	1	1	1	1	0	1	1	1	8	Low
Head et al. (2006) [32]	1	1	1	1	0	1	1	0	1	7	Low
Olek et al. (2023) [23]	1	1	1	1	1	1	1	1	1	9	Low
Olek et al. (2024) [24]	1	1	1	1	1	1	1	1	1	9	Low
Sharma et al. (2012) [33]	1	1	1	0	0	1	1	0	1	6	Moderate
Laffers et al. (2015) [34]	1	1	1	1	0	1	1	0	0	6	Moderate
Wozniak et al. (2023) [35]	1	1	1	1	0	1	1	0	1	7	Low
Xu et al. (2010) [25]	1	1	1	1	1	1	1	1	1	9	Low

**Table 4:** A general overview of the studies.

Author and Year	Country	Study Design
Bhuvaneswari et al. (2007) [26]	Singapore	In vivo study
Blank et al. (2001) [27]	Israel	In vivo study
Bublik et al. (2006) [28]	USA	In vitro study
Du et al. (2002) [29]	Singapore	In vitro study
Du et al. (2003) [30]	Singapore	In vivo study
Du et al. (2004) [31]	Singapore	In vitro study
Head et al. (2006) [32]	USA	In vitro study
Olek et al. (2023) [23]	Poland	In vitro study
Olek et al. (2024) [24]	Poland	In vitro study
Sharma et al. (2012) [33]	South Africa	In vitro study
Laffers et al. (2015) [34]	Germany	In vitro study
Wozniak et al. (2023) [35]	Poland	In vitro study
Xu et al. (2010) [25]	China	In vitro study

**Table 5:** Main outcomes and details from each study.

Study Authors	Cancer Cell Type	Focus	Mechanisms Explored	Outcomes	Treatment Related Adverse Events
Bhuvaneswari et al. (2007) [26]	HK1, CNE-2	VEGF expression post-PDT	Photodynamic therapy induces hypoxia within tumors, which triggers the expression of VEGF through the HIF-1α pathway. The use of celebrex (a COX-2 inhibitor) to modulate VEGF expression post-PDT.	VEGF levels are initially downregulated post-PDT but are upregulated within 72 h, indicating tumor regrowth. Combination therapy with celebrex significantly downregulates VEGF expression, potentially improving PDT outcomes.	
Blank et al. (2001) [27]	Highly invasive solid tumors; DA3Hi mammary adenocarcinoma and SQ2 squamous cell carcinoma.	Evaluating the tumoricidal effects of HY-PDT on primary tumor development, survival rates, and metastatic spread in mice.	HY-PDT induces extensive tumor necrosis and inflammation but not significant immune antitumoral responses. The therapy stimulates the expression of inflammatory cytokines (e.g., IL-1β, IL-6, TNF-α) in tumor tissues and systemically in the spleen. No significant effect on immune-related cytokine mRNAs, such as IL-2, IL-4, and IFN-γ, was observed.	HY-PDT delayed tumor development and prolonged survival in mice when applied to smaller tumors. It was more effective on SQ2 squamous cell carcinoma compared with DA3Hi adenocarcinoma. Slight reductions in metastatic burden were observed in SQ2-bearing mice, but no significant effects were noted in DA3Hi-bearing mice. HY-PDT induced extensive tumor necrosis, accompanied by local and systemic inflammatory responses, as evidenced by elevated mRNA levels of inflammation-related cytokines (e.g., IL-1β, IL-6, TNF-α). However, no antitumoral immune responses were observed.	Limited systemic immune responses and an absence of HY-PDT-induced antitumoral immunity. Potential for localized tissue damage and inflammatory reactions at treatment sites.
Bublik et al. (2006) [28]	HNSCC	Evaluating HY-PDT using pulsed laser light at various wavelengths to determine optimal conditions for phototoxicity and tumor targeting in vitro.	Hypericin is activated by laser light to generate singlet oxygen and reactive species, leading to tumor cell death. Light absorption peaks at 545 and 595 nm, with 593 nm being the optimal wavelength for phototoxicity. Picosecond pulsed laser light is more effective than millisecond pulses due to higher intensity and deeper tissue penetration. Hypericin localizes predominantly in the perinuclear region, affecting the endoplasmic reticulum and Golgi apparatus.	Hypericin absorbs light at 545 and 595 nm and emits fluorescence at 594 and 640 nm, with its tricyclic structure enabling the production of singlet oxygen, which is crucial for PDT. In vitro studies on SCC show that phototoxicity increases with hypericin concentration, exposure time, and laser power, with a significant linear increase in cell toxicity observed at higher drug doses and light fluence. Phototoxicity was enhanced at wavelengths near hypericin’s absorption peaks, with 550 nm light showing similar effectiveness to 514 nm light but requiring less energy. The optimal wavelength for PDT was found to be 593 nm, where minimal energy was required for maximum tumor toxicity. Picosecond laser pulses induced greater tumor cell cytotoxicity compared with millisecond pulses, even with equal energy delivery, suggesting that the higher intensity of shorter pulses is more effective. Confocal microscopy revealed that hypericin accumulates in the perinuclear region of SCC cells, leading to rapid cytotoxic effects, such as cell blebbing, upon light exposure, confirming its potential for inducing fluorescence and tumor phototoxicity under both visible and infrared light activation.	Limited systemic toxicity observed, but specific adverse events were not detailed as this was an in vitro study.
Du et al. (2002) [29]	NPC/HK1	Investigating the endogenous production of cytokines (IL-8 and IL-10) in two EBV-positive NPC cell lines (HK1 and CNE-2) and assessing the effects of hypericin and hypericin-mediated photodynamic therapy on these cytokines.	PDT is known to upregulate IL-8 transcription via ROS and activate the IL-10 promoter.	IL-8 was constitutively expressed in both cell lines; levels were 2-fold higher in HK1 compared with CNE-2 (*p* = 0.0004). Hypericin increased IL-8 by almost 30% in HK1 cells (*p* = 0.0180). IL-10 was undetectable in all conditions. HY-PDT did not significantly alter IL-8 or IL-10 levels in either cell line. Cytokine responses varied between cell lines, highlighting tumor microenvironment differences.	No specific adverse effects linked to cytokine production were reported.
Du et al. (2003) [30]	NPC/HK1	Evaluation of the efficacy of HY-PDT for treating NPC, emphasizing the relationship between hypericin biodistribution and photodynamic effects.	Biodistribution of hypericin: Rapid plasma peak concentration at 1 h post-administration, with maximal tumor uptake at 6 h. Tumor shrinkage mechanisms: Combination of vascular damage and direct tumor cell killing. Inflammatory response: Prominent neutrophil infiltration and intratumoral hemorrhage in PDT-treated tumors. Fluorescence properties: Hypericin absorption peaks at 470, 545, and 595 nm; fluorescence emission peaks at 590 and 640 nm.	Maximal tumor regression observed when light irradiation occurred 6 h post-hypericin injection. Comparable tumor RRP observed at 1 h and 6 h PDT intervals (*p* = 0.122).PDT at all intervals significantly inhibited tumor growth compared with controls (*p* < 0.001). Tumor necrosis, morphological changes, and significant inflammatory cell infiltration were evident in PDT-treated tumors. No anti-tumor effects with hypericin or light alone.	Not explicitly reported in the provided text.
Du et al. (2004) [31]	HK1 CNE-2 NPC cells	Evaluation of the effect of HY-PDT on GST activity in NPC cells.	HY-PDT induces ROS, including superoxide anion radicals and hydroxyl radicals. ROS-mediated oxidative stress downregulates GST activity. Impact of reduced GST activity on cell viability and tumor response to PDT.	Significant reduction in GST activity in vitro (HK1: 27% of baseline; CNE-2: 60% of baseline). In vivo GST activity in HK1 tumors significantly decreased at 16 and 24 h post-PDT. PDT induced 69% and 53% cell death in HK1 and CNE-2 cells, respectively.	Not reported in the study.
Head et al. (2006) [32]	HNSCC	Evaluating HY-PDT for tumor imaging and treatment, optimizing conditions for phototoxicity in vitro and testing its application in vivo in a mouse model.	Hypericin is activated by visible and near-infrared laser light to generate singlet oxygen and reactive oxygen species, leading to tumor cell death. Optimal phototoxic effects were observed at a laser wavelength of 593 nm, corresponding to hypericin’s absorption maximum. Hypericin localized in tumors, remaining effective for up to 10 days post-injection, as confirmed by fluorescence imaging using fiberoptic lasers.	In vitro HY-PDT showed a dose–response relationship, with significant tumoricidal effects at 0.2–0.5 μg/mL and enhanced cytotoxicity at 593 nm with 150 mW laser power. In vivo: Tumors under 0.4 cm^2^ responded well to biweekly hypericin PDT, showing regression. Larger tumors exhibited partial response or regrowth, highlighting the limitations of light penetration at 532 nm. The study suggested hypericin as a valuable agent for defining and sterilizing tumor margins during resection.	While the study reported no systemic toxicity, it highlighted challenges with light penetration in larger tumors, necessitating advancements in laser technology or treatment strategies.
Olek et al. (2023) [23]	OSCC	Investigating the immunomodulatory effects of HY-PDT on cancer cells (SCC-25) and healthy gingival fibroblasts (HGF-1).	HY-PDT employs light-activated hypericin to induce oxidative stress via reactive oxygen species, leading to cell death. HY-PDT modulates cytokine secretion, affecting inflammatory and immunosuppressive pathways. Specific cytokines (e.g., IL-6, IL-8, IL-20, PTX3) and soluble receptors (e.g., sIL-6R) were evaluated for their response to PDT.	HY-PDT demonstrated cytotoxicity toward both cancer cells and fibroblasts, starting at a light dose of 5 J/cm^2^ and increasing with higher doses. Cytokine analysis revealed significant alterations, as follows: Increased secretion of IL-20 and sIL-6Rbeta in cancer cells following HY-PDT, enhanced IL-8 secretion with hypericin alone (no irradiation) for both cell lines, and reduced PTX3 secretion post-PDT in cancer cells. HY-PDT did not significantly alter IL-6 or IL-10 secretion.	Lack of selectivity for cancer cells, with observed cytotoxicity toward healthy fibroblasts, indicating potential for off-target effects.
Olek et al. (2024) [24]	OSCC SCC-25	Investigating the effects of HY-PDT on the secretion of soluble TNF receptors (sTNF-R1 and sTNF-R2) by SCC-25 and healthy gingival fibroblasts (HGF-1).	HY-PDT generates cytotoxic effects via reactive oxygen species and modulates immune responses. The role of TNF-α signaling and its soluble receptors in inflammation and immune modulation were examined. Secretion of soluble TNF-α receptors was measured after sublethal PDT doses in order to understand immunomodulatory effects.	HY-PDT increased sTNF-R1 secretion by SCC-25 after sublethal doses, with no effect on sTNF-R1 production in fibroblasts. PDT had no effect on sTNF-R2 secretion in either cell line. Cytotoxic effects of HY-PDT were dependent on the dose of hypericin and light.	PDT-induced cytotoxicity was not selective for cancer cells, indicating potential harm to healthy tissues at higher doses.
Sharma et al. (2012) [33]	Non-melanoma cutaneous SCC	Investigating the efficacy of HY-PDT and its mode of cell death in SCC cell cultures, with a focus on optimizing treatment through a “double-hit” (two-day) strategy.	Hypericin is activated by UV light (320–400 nm) to produce ROS, leading to tumor cell death. Cell death was primarily necrotic and caspase-independent, differing from apoptosis. ROS levels peaked after the first day of treatment and decreased on the second day.	A significant dose-dependent reduction in SCC cell viability was observed after two days of HY-PDT treatment. Necrotic cell death was associated with increased ROS production on the first day. The “double-hit” treatment strategy was more effective in reducing cell viability compared with a single treatment.	Potential activation of inflammatory mediators due to necrotic cell death, which might contribute to tumor-specific immunity, although this was not directly measured in the study.
Laffers et al. (2015) [34]	HNSCC—cell line FaDu	Investigating HY-PDT on HNSCC cells (FaDu cell line) in vitro, focusing on metabolic activity and apoptotic pathways.	Hypericin accumulates in tumor cells and is activated by light (450–700 nm), generating ROS. Hypericin-mediated PDT induces apoptosis and/or necrosis through caspase-dependent and independent pathways. Activation of hypericin results in damage to mitochondria, endoplasmic reticulum, and Golgi apparatus, initiating cell death.	FaDu cells treated with hypericin (5–50 µM) and illuminated for 10–25 min showed a significant reduction (92–97%) in metabolic activity after 1–8 days. Apoptosis was detected in nearly all cells treated with hypericin and light, with no apoptosis observed in untreated or non-illuminated cells. Higher hypericin concentrations and longer light exposure did not yield significantly greater effects, indicating efficiency at low doses and short exposure.	Hypericin treatment requires light activation; no dark toxicity was observed. Potential inflammation due to necrosis was not assessed in detail but is a consideration for future studies.
Wozniak et al. (2023) [35]	SCC-25 cells and MUG-Mel2	Investigating the selectivity and phototoxic effects of HY-PDT on melanoma (MUG-Mel2) and squamous cell carcinoma (SCC-25) compared with normal keratinocytes (HaCaT).	Hypericin is activated by orange light (590 nm), producing ROS that lead to cytotoxic effects. Cellular uptake of hypericin was assessed, showing selective accumulation in cancer cells. Apoptosis induction was evaluated using TUNEL assays and morphological changes.	PDT with hypericin showed higher phototoxicity in cancer cells (MUG-Mel2 and SCC-25) than in normal keratinocytes. A dose of 1 µM hypericin combined with orange light irradiation significantly reduced viability, as follows: MUG-Mel2: 21% cell viability; SCC-25: 20% cell viability; HaCaT: 26% cell viability. Apoptosis was observed in 52% of MUG-Mel2 cells and 23% of SCC-25 cells post-PDT. Morphological analysis revealed apoptotic changes such as cell rounding and detachment.	Minimal phototoxicity was noted in normal cells compared with cancer cells, suggesting a promising therapeutic window. Limitations include hypericin’s poor solubility and sensitivity to environmental factors, which may affect its application.
Xu et al. (2010) [25]	NPC/ CNE-2 cells	Evaluating the efficacy of HY-PDT in inducing cell destruction and apoptosis in CNE-2 cells.	Hypericin is activated by red light (590 nm) to generate ROS, leading to apoptosis and cell death. The study explored early and late apoptosis using Hoechst staining for nuclear changes and flow cytometry with annexin V and PI. Two apoptotic pathways were considered: mitochondria-dependent and death receptor-dependent.	HY-PDT resulted in dose-dependent cytotoxicity based on both drug concentration (0–2.5 μM) and light fluence (1–8 J/cm^2^). Early apoptosis was identified as the primary mode of cell death, with an early apoptotic rate of 53.08% and late apoptosis at 6.77%. Cellular destruction included membrane blebbing, cell shrinkage, and nuclear condensation.	No significant cytotoxicity was observed in the absence of light, indicating hypericin’s safety without photoactivation.

HNSCC: head and neck squamous cell carcinoma; OSCC: oral squamous cell carcinoma; SCC: squamous cell carcinoma; NPC: nasopharyngeal carcinoma; PDT: photodynamic therapy; ROS: reactive oxygen species; PI: propidium iodide; HY-PDT: hypericin-mediated photodynamic therapy; HIF-1α: hypoxia-inducible factor 1-alpha; RRP: relative regression percentage; TNF: tumour necrosis factor; GST: glutathione S-transferase; VEGF: vascular endothelial growth factor; COX-2: cyclooxygenase-2; IL: interleukin; TNF-α: tumour necrosis factor alpha; sTNF-R1/2: soluble tumour necrosis factor receptor 1/2; sIL-6R: soluble interleukin-6 receptor; IFN-γ: interferon gamma; EBV: Epstein–Barr virus; TUNEL: terminal deoxynucleotidyl transferase dUTP nick end labelling; mRNA: messenger ribonucleic acid; J/cm^2^: Joules per square centimetre.

**Table 6:** Physical parameters of light sources.

Author/Year	Light Source	Operating Mode	Wavelength (nm)	Energy Density (Fluence) (J/cm^2^)	Power Output (mW)	Powermeter Used	Irradiation Time (s)
Bhuvaneswari et al. (2007) [26]	Halogen light source (Zeiss KL1500)	Filtered bandpass light	560–640	120	50	Yes	Not specified
Blank et al. (2001) [27]	Polychromatic visible light	Not specified	560	60	Not specified	Not specified	20 min
Bublik et al. (2006) [28]	Pulsed dye laser Ti:Sapphire laser	Pulsed, two-photon	514, 550, 593	1–9	50, 100, 150	Not specified	0–120
Du et al. (2002) [29]	Fluorescence tubes (Phillips type OSRAM L30w11–860) with acetate filter	Wide illumination band	Above 585	0.5	30	Not specified	Not specified
Du et al. (2003) [30]	Halogen lamp with red acetate filter	Wide illumination band	Above 590	120	360	Yes	Not specified
Du et al. (2004) [31]	In vitro: Bank of fluorescent tubes (Phillips type OSRAM L30w11–860, 30 W) In vivo: Halogen lamp (360 W, Osram, Mexico)	Continuous mode	Above 585	In vitro: 0.5 J/cm^2^ In vivo: 120 J/cm^2^	226	Not specified	Not specified
Head et al. (2006) [32]	KTP532 laser	Green light, fiberoptic delivery	532, 550, 593	0–60	50, 100, 150	Not specified	0–120
Olek et al. (2023) [23]	TP-1 PDT lamp	Orange and infrared light filters	580–720	0–20	35 mW/cm^2^.	Not specified	Automatically controlled
Olek et al. (2024) [24]	PDT TP1 photodynamic lamp	Incoherent	580–720	0, 1, 2, 5, 10, 20	35 mW/cm^2^.	Not specified	Automatically controlled
Sharma et al. (2012) [33]	PUVA lamps (F15W/T8)	Continuous	315–400	1	Not specified	Not specified	Not specified
Laffers et al. (2015) [34]	HQI^®^-TS lamp (Osram)	450–700 nm spectrum	450, 548, ~600	Not specified	50,000 lx	Not specified	0, 600, 1500
Wozniak et al. (2023) [35]	Halogen lamp (Penta lamps)	Orange light	590	3.6, 7.2	120	Not specified	30, 60
Xu et al. (2010) [25]	400-watt quartz-halogen lamp	590 nm long-pass filter	590	1–8	8	Yes	Not specified

**Table 7:** Characteristics of PS used in studies meeting eligibility criteria.

Author and Year	Incubation Time (Minutes)	Concentration/s of Hypericin Used
Bhuvaneswari et al. (2007) [26]	360	5 mg/mL
Blank et al. (2001) [27]	30	Not specified
Bublik et al. (2006) [28]	60	0.05 to 1 µg/mL
Du et al. (2002) [29]	HK1: 240 CNE-2: 360	0.5, 1 µM
Du et al. (2003) [30]	Not specified	1 mg/mL
Du et al. (2004) [31]	HK1: 240 CNE-2: 360	In vitro: 0.5 µM In vivo: 2 mg/kg
Head et al. (2006) [32]	60	Varied micromolar range
Olek et al. (2023) [23]	120	0, 0.25, 0.5, 1 µM
Olek et al. (2024) [24]	120	0, 0.25, 0.5, 1 µM
Sharma et al. (2012) [33]	240	0–7 mM
Laffers et al. (2015) [34]	150	0, 5, 10, 25, and 50 μM
Wozniak et al. (2023) [35]	120	0.1, 0.5, 1, 2.5, 5 µM
Xu et al. (2010) [25]	120	0–2.5 μM

## Data Availability

Not applicable.

## References

[1] Argiris A., Karamouzis M.V., Raben D., Ferris R.L. (2008). Head and neck cancer. Lancet.

[2] Raber-Durlacher J.E., Brennan M.T., Leeuw I.M.V.-D., Gibson R.J., Eilers J.G., Waltimo T., Bots C.P., Michelet M., Sollecito T.P., Rouleau T.S. (2012). Swallowing dysfunction in cancer patients. Care Cancer.

[3] Baskar R., Lee K.A., Yeo R., Yeoh K.W. (2012). Cancer and radiation therapy: Current advances and future directions. Int. J. Med. Sci..

[4] Rueda J.R., Solà I., Pascual A., Subirana Casacuberta M. (2011). Non-invasive interventions for improving well-being and quality of life in patients with lung cancer. Cochrane Database Syst. Rev..

[5] Kim T.E., Chang J.E. (2023). Recent Studies in Photodynamic Therapy for Cancer Treatment: From Basic Research to Clinical Trials. Pharmaceutics.

[6] Correia J.H., Rodrigues J.A., Pimenta S., Dong T., Yang Z. (2021). Photodynamic Therapy Review: Principles, Photosensitizers, Applications, and Future Directions. Pharmaceutics.

[7] Aebisher D., Woźnicki P., Bartusik-Aebisher D. (2024). Photodynamic Therapy and Adaptive Immunity Induced by Reactive Oxygen Species: Recent Reports. Cancers.

[8] Thiruppathi J., Vijayan V., Park I.K., Lee S.E., Rhee J.H. (2024). Enhancing cancer immunotherapy with photodynamic therapy and nanoparticle: Making tumor microenvironment hotter to make immunotherapeutic work better. Front. Immunol..

[9] Wang X., Wang L., Fekrazad R., Zhang L., Jiang X., He G., Wen X. (2023). Polyphenolic natural products as photosensitizers for antimicrobial photodynamic therapy: Recent advances and future prospects. Front. Immunol..

[10] Brancaleon L., Moseley H. (2002). Laser and non-laser light sources for photodynamic therapy. Lasers Med. Sci..

[11] Kim M.M., Darafsheh A. (2020). Light Sources and Dosimetry Techniques for Photodynamic Therapy†. Photochem. Photobiol..

[12] Buytaert E., Callewaert G., Hendrickx N., Scorrano L., Hartmann D., Missiaen L., Vandenheede J.R., Heirman I., Grooten J., Agostinis P. (2006). Role of endoplasmic reticulum depletion and multidomain proapoptotic BAX and BAK proteins in shaping cell death after hypericin-mediated photodynamic therapy. FASEB J. Off. Publ. Fed. Am. Soc. Exp. Biol..

[13] Mansoori B., Mohammadi A., Amin Doustvandi M., Mohammadnejad F., Kamari F., Gjerstorff M.F., Baradaran B., Hamblin M.R. (2019). Photodynamic therapy for cancer: Role of natural products. Photodiagnosis Photodyn. Ther..

[14] Deng B., Wang K., Zhang L., Qiu Z., Dong W., Wang W. (2023). Photodynamic Therapy for Inflammatory and Cancerous Diseases of the Intestines: Molecular Mechanisms and Prospects for Application. Int. J. Biol. Sci..

[15] Kciuk M., Yahya E.B., Mohamed M.M.I., Rashid S., Iqbal M.O., Kontek R., Abdulsamad M.A., Allaq A.A. (2023). Recent Advances in Molecular Mechanisms of Cancer Immunotherapy. Cancers.

[16] Cha J.H., Chan L.C., Song M.S., Hung M.C. (2020). New Approaches on Cancer Immunotherapy. Cold Spring Harb. Perspect. Med..

[17] Edwards S.C., Hoevenaar W.H.M., Coffelt S.B. (2021). Emerging immunotherapies for metastasis. Br. J. Cancer.

[18] Mirza F.N., Khatri K.A. (2017). The use of lasers in the treatment of skin cancer: A review. J. Cosmet. Laser Ther..

[19] Schardt C., Adams M.B., Owens T., Keitz S., Fontelo P. (2007). Utilization of the PICO Framework to Improve Searching PubMed for Clinical Questions. BMC Med. Inform. Decis. Mak..

[20] Page M.J., McKenzie J.E., Bossuyt P.M., Boutron I., Hoffmann T.C., Mulrow C.D., Shamseer L., Tetzlaff J.M., Akl E.A., Brennan S.E. (2021). The PRISMA 2020 Statement: An Updated Guideline for Reporting Systematic Reviews. BMJ.

[21] Watson P.F., Petrie A. (2010). Method Agreement Analysis: A Review of Correct Methodology. Theriogenology.

[22] Higgins J., Thomas J., Chandler J., Cumpston M., Li T., Page M. (2023). Welch Cochrane Handbook for Systematic Reviews of Interventions Version 6.4.

[23] Olek M., Machorowska-Pieniążek A., Czuba Z.P., Cieślar G., Kawczyk-Krupka A. (2023). Effect of hypericin-mediated photodynamic therapy on the secretion of soluble TNF receptors by oral cancer cells. Pharmaceutics.

[24] Olek M., Machorowska-Pieniążek A., Czuba Z.P., Cieślar G., Kawczyk-Krupka A. (2024). Immunomodulatory effect of hypericin-mediated photodynamic therapy on oral cancer cells. Pharmaceutics.

[25] Xu C.S., Leung A.W.N. (2010). Light-activated hypericin induces cellular destruction of nasopharyngeal carcinoma cells. Laser Phys. Lett..

[26] Bhuvaneswari R., Gan Y.Y.-Y., Yee K.K.L., Soo K.C., Olivo M. (2007). Effect of hypericin-mediated photodynamic therapy on the expression of vascular endothelial growth factor in human nasopharyngeal carcinoma. Int. J. Mol. Med..

[27] Blank M., Lavie G., Mandel M., Keisari Y. (2001). Effects of photodynamic therapy with hypericin in mice bearing highly invasive solid tumors. Oncol. Res..

[28] Bublik M., Head C., Benharash P., Paiva M., Eshraghi A., Kim T., Saxton R. (2006). Hypericin and pulsed laser therapy of squamous cell cancer in vitro. Photomed. Laser Surg..

[29] Du H., Bay B.H., Mahendran R., Olivo M. (2002). Endogenous expression of interleukin-8 and interleukin-10 in nasopharyngeal carcinoma cells and the effect of photodynamic therapy. Int. J. Mol. Med..

[30] Du H.Y., Bay B.H., Olivo M. (2003). Biodistribution and photodynamic therapy with hypericin in a human NPC murine tumor model. Int. J. Oncol..

[31] Du H.Y., Olivo M., Tan B.K., Bay B.H. (2004). Photoactivation of hypericin down-regulates glutathione S-transferase activity in nasopharyngeal cancer cells. Cancer Lett..

[32] Head C.S., Luu Q., Sercarz J., Saxton R. (2006). Photodynamic therapy and tumor imaging of hypericin-treated squamous cell carcinoma. World J. Surg. Oncol..

[33] Sharma K.V., Davids L.M. (2023). Hypericin-PDT-induced rapid necrotic death in human squamous cell carcinoma cultures after multiple treatments. Redox Lab..

[34] Laffers W., Busse A.-C., Mahrt J., Nguyen P., Gerstner A.O.H., Bootz F., Wessels J.T. (2015). Photosensitizing effects of hypericin on head neck squamous cell carcinoma in vitro. Eur. Arch. Oto-Rhino-Laryngol..

[35] Wozniak M., Nowak-Perlak M. (2023). Hypericin-based photodynamic therapy displays higher selectivity and phototoxicity towards melanoma and squamous cell cancer compared to normal keratinocytes in vitro. Int. J. Mol. Sci..

[36] Dong X., Dong X., Zeng Y., Zeng Y., Zhang Z., Zhang Z., Fu J., Fu J., You L., You L. (2021). Hypericin-mediated photodynamic therapy for the treatment of cancer: A review. J. Pharm. Pharmacol..

[37] Kamuhabwa A.R., Agostinis P.M., D’Hallewin M.-A., Baert L., de Witte P.A.M. (2001). Cellular photodestruction induced by hypericin in AY-27 rat bladder carcinoma cells. Photochem. Photobiol..

[38] Zupko I., Kamuhabwa A.R., D’Hallewin M.-A., Baert L., De Witte P.A. (2001). In vivo photodynamic activity of hypericin in transitional cell carcinoma bladder tumors. Int. J. Oncol..

[39] Huygens A., Kamuhabwa A.R., Van Laethem A., Roskams T., Van Cleynenbreugel B., Van Poppel H., Agostinis P., De Witte P.A. (2005). Enhancing the photodynamic effect of hypericin in tumour spheroids by fractionated light delivery in combination with hyperoxygenation. Int. J. Oncol..

[40] Blank M., Kostenich G., Lavie G., Kimel S., Keisari Y., Orenstein A. (2002). Wavelength-dependent properties of photodynamic therapy using hypericin in vitro and in an animal model. Photochem. Photobiol..

[41] Ferenc P., Solár P., Kleban J., Mikeš J., Fedoročko P. (2010). Down-regulation of Bcl-2 and Akt induced by combination of photoactivated hypericin and genistein in human breast cancer cells. J. Photochem. Photobiol. B Biol..

[42] Delaey E.M., Vandenbogaerde A.L., Agostinis P., De Witte P.A. (1999). Confluence dependent resistance to photo-activated hypericin in HeLa cells. Int. J. Oncol..

[43] Miccoli L., Beurdeley-Thomas A., De Pinieux G., Sureau F., Oudard S., Dutrillaux B., Poupon M.F. (1998). Light-induced photoactivation of hypericin affects the energy metabolism of human glioma cells by inhibiting hexokinase bound to mitochondria. Cancer Res..

[44] Uzdensky A.B., Ma L.W., Iani V., Hjortland G.O., Steen H.B., Moan J. (2001). Intracellular localisation of hypericin in human glioblastoma and carcinoma cell lines. Lasers Med. Sci..

[45] Lavie G., Kaplinsky C., Toren A., Aizman I., Meruelo D., Mazur Y., Mandel M. (1999). A photodynamic pathway to apoptosis and necrosis induced by dimethyl tetrahydroxyhelianthrone and hypericin in leukaemic cells: Possible relevance to photodynamic therapy. Br. J. Cancer.

[46] Liu C.D., Kwan D., Saxton R.E., McFadden D.W. (2000). Hypericin and photodynamic therapy decreases human pancreatic cancer in vitro and in vivo. J. Surg. Res..

[47] Chen B., Roskams T., Xu Y., Agostinis P., de Witte P. (2002). Photodynamic therapy with hypericin induces vascular damage and apoptosis in the RIF-1 mouse tumor model. Int. J. Cancer.

